# Analysis of prescription database extracted from standard textbooks of traditional Dai medicine

**DOI:** 10.1186/1746-4269-8-34

**Published:** 2012-08-29

**Authors:** Chuang Zhang, Virasakdi Chongsuvivatwong, Niwat Keawpradub, Yanfang Lin

**Affiliations:** 1 Faculty of Ethnomedicine, Yunnan University of Traditional Chinese Medicine, No. 1076 Yuhua Road, Chenggong, Kunming, Yunnan Province, 650500, China; 2 Epidemiology Unit, Faculty of Medicine, Prince of Songkla University, HatYai, Songkhla, 90110, Thailand; 3 Faculty of Pharmacy, Prince of Songkla University, HatYai, Songkhla, 90110, Thailand; 4 Xishuangbanna Dai Medical Hospital, No.17 Minhang Road, JingHong, Yunnan, 666100, China

**Keywords:** Traditional Dai medicine, Dai medical textbooks, Dai prescription

## Abstract

**Background:**

Traditional Dai Medicine (TDM) is one of the four major ethnomedicine of China. In 2007 a group of experts produced a set of seven Dai medical textbooks on this subject. The first two were selected as the main data source to analyse well recognized prescriptions.

**Objective:**

To quantify patterns of prescriptions, common ingredients, indications and usages of TDM.

**Methods:**

A relational database linking the prescriptions, ingredients, herb names, indications, and usages was set up. Frequency of pattern of combination and common ingredients were tabulated.

**Results:**

A total of 200 prescriptions and 402 herbs were compiled. Prescriptions based on "wind" disorders, a detoxification theory that most commonly deals with symptoms of digestive system diseases, accounted for over one third of all prescriptions. The major methods of preparations mostly used roots and whole herbs.

**Conclusion:**

The information extracted from the relational database may be useful for understanding symptomatic treatments. Antidote and detoxification theory deserves further research.

## Background

### Dai minority and traditional Dai medicine

In China, traditional Dai Medicine (TDM), one system of ancient ethnomedicine, has accumulated more than 2,500 years of experience in the process of struggling with the treatment of diseases [1]. It has absorbed and integrated practices from Indian medicine, Hinayana Buddhism and local wisdom to form a unique medical theory. In 1984, the government of China defined TDM as one of China's four important systems of ethnomedicine. The other three are from Tibetan, Mongolian and Uygur origins [2].

Dai people have their own unique language which belongs to the Tai-Lao family. Its alphabet is derived from the ancient Indian alphabet. Dai people mainly inhabit the southern part of Yunnan province of China, but there are significant populations living also in Myanmar, Laos, northern Thailand and Vietnam.

In the past, the main-stream culture and education was obtained through temples. The temple-based education system maintains and integrates religion, written language, values, native perception, mythology and medicine. The inheritance of TDM has two different ways. One is passing down from generation to generation by oral communication and learned by heart. The other is for the literate people learning from documents such as “Tam Lan” (the sutra carved on the palm leaves by Buddhists), which are the media for both TDM and Hinayana Buddhism [3].

Although TDM has accumulated a wealth of medical knowledge and related works, it is gradually vanishing due to the impact and prevalence of western medicine and modern hospitals [3].

In 2004, in order to preserve TDM and train more Dai medical professionals, the State Administration of Traditional Chinese Medicine (TCM) of China set up a project named “21^st^ century general higher educational teaching material of TDM” and entrusted the Yunnan Provincial Department of Health to organize relevant units and experts to compile it. Famous experts (“Famous” is a high ranking title) and professors in related fields throughout China were invited to form an academic council, the members of which included the last king of Dai nationality, Famous Old Traditional Dai doctors (TDD), academicians of pharmacy and Famous TCM doctors. Finally, the Ethnomedicine Research and Development Center of Yunnan University of TCM and Xishuangbanna Dai Medical Hospital coordinated and completed the project. Consequently since 2007, a set of seven Dai medical textbooks, published by Chinese Publishing House of TCM, has been used as the general higher educational teaching materials for the undergraduates whose major is TDM. The textbooks include *Formulas of Dai Medicine* (Book A) [4], *Dai Materia Medica* (Book B) [5], *Basic Theories of Dai Medicine* (Book C) [6], *Diagnostics of Dai Medicine* (Book D) [7], *Clinical Dai Medicine, History of Dai Medicine* and *Selected Readings of Dai Medicinal Classic*. This first set of Dai medical undergraduate textbooks is the most authoritative Dai medical monographs of TDM of China at present.

### Brief introduction to traditional Dai medicine theory

TDM theory has its own unique characteristics. “Ta Du Dang Si” Theory (TDDST or caturmahabhuta) and “Han Ta Dang Ha” Theory (HTDHT or skandha) are the core theories. TDDST regards the body as being formed by four cosmic elements including wind, fire, water and earth. Disharmony of these elements gives rise to pain and disease while harmony results in good health. HTDHT refers to form, consciousness, feeling, perception and intention to explain the physiological structure, phenomena and spiritual activities of the body [1,6].

“Ya Gei” theory (YGT or Detoxification theory) is a unique supplementary theory of TDM. Its core content is to “detoxify before a disease commences and treat a disease after detoxification". TDM believes that all materials which can enter the human body, including food, can produce certain poisonous effects to the body if they are consumed excessively. In order to keep healthy, people must take antidotes regularly to eliminate these micro toxins, and thus reduce the chance of illness and prolong life [6].

Therefore, Dai medicinal theory believes that in the process of daily life, all kinds of internal and external pathogenic factors may lead to imbalance of four cosmic elements and produce some toxins, consequently generating disorders of physiological functions. However, antidotes could be used for release of toxins and adjustments of physiological functions. Therefore, keep the balance of functions of four elements can reduce illness and death [6].

The fact that prescription data are systematically recorded in the books affords us an opportunity to analyse and gain better insights into how TDM makes use of the existing herbs in their medicine. The objectives of this study are to extract and analyse main information from these standard textbooks and quantify the patterns of prescriptions, indications and preparations.

## Materials and methods

### Data source

Books A and B were related to prescriptions and herbs, so these were selected as the main data source. Books C and D did not contribute to the database, but could provide terminology, concept and theory, and were therefore used in explanation of the relationship among variables in the database.

### Prescriptions

Each prescription involves the name, ingredients, indications, dosage and method of preparation. The names were already transliterated and paraphrased into the Han (Chinese) language.

### Indications

The indications of prescriptions are described with either modern disease names, or syndrome and symptoms. We did initial verbatim translation to English. However, it was obvious that certain diseases were interpreted by an editor with a western medicine concept, e.g. urinary disease, immune disease etc. The disease names were therefore omitted. Only the indications such as common symptoms and syndromes were included.

### Ingredients

The names of the ingredients were listed both in Dai and Han. Regardless of the availability of the botanical names, all ingredient names were put into the database.

### Plant parts

If the information on plant parts was not available in Book A we used the information about plant parts recorded in Book B which was a monograph of Dai medicinal plants.

### Coding and computerization

Each indication/symptom/disease name/ herb/ medicinal part was given a unique code according to the following principles:

1) Synonyms were given the same code

2) Redundant or non-symptom information was deleted

3) Long descriptive words were simplified

After data entry by the first author, two other persons randomly checked 40% of the selected data to minimize the errors.

### Relational structure of the database

The different components of herbs could also be used in multiple prescriptions, preparations and dosages. The information was computerized in a relational database. The major fields included prescriptions name, composition, herbs, indications, medicinal parts, attributions, and usages.

### Data analysis

The frequency/proportion of medicinal parts and preparations was summarized by tabulation. The frequency of occurrence of herbs, prescriptions and indications was calculated in percentage. Only those with relatively high frequencies were selected and listed in tables in this article.

## Results and discussion

### Prescriptions, preparations and attributions

According to TDM theory from Books C and D, components of Dai prescriptions can be categorized into principal ingredients, assistant ingredients, inhibiting ingredients, harmonizing ingredients and efficacy-enhancing ingredients. According to their different roles and effects in the prescriptions, they can restrain each other and, therefore, enhance the impact and reduce the side effects and toxicity. In addition, both ingredients and prescriptions have different attributions, and each of them at least belongs to one element, so they could be used to against specific symptoms or disorders [4]. After reasonable combination, they could be used to rectify the excesses or deficiencies of these elements in the body. For example, the diseases belonging to wind disorder are the most complex and extensive because wind can stimulate the human reproductive development, and promote metabolism, digestion and absorption of food and excretion. So, digestive system diseases, rheumatism and joint pain all belong to wind element disorders and can be cured by the herbs or prescriptions belonging to wind [6].

In Book A, 200 classic prescriptions are encompassed, including treatments of all four element disorders (8), wind disorders (40), fire disorders (40), water disorders (40), earth disorders (40), antidotes (16), and others (16). All prescriptions have more than one herb. Table 1 displays 11 selected prescriptions with the highest number of clinical indications, 5 of them belonging to antidote, 4 to wind element and 2 to fire attributions.

**Table 1:** The top eleven most widely applied prescriptions

**Prescription name**	**Number of indications**	**Attribution**
Ya-Peng-Leng	10	Wind
Ya-Peng-Lang	9	Wind
Ya-Gei-Shuang-Long	8	Antidote
Fei-Mai-Nan-Huang-Luo	8	Antidote
Ya-Gei-Na-Er	7	Fire
Ya-Gei-Sa-Ba	7	Antidote
Ya-Gei-Bi	7	Antidote
Ya-Nuan-Long-Lan-Shen	6	Wind
Ya-Dong-Hong	6	Wind
Ya-Sha-Long-Jie-Huo	6	Fire
Ya-Xi-Li-Meng-La-Ga-Han	6	Antidote

Table 2 shows the relationship between attributions and preparations. Decoction contributes more than 60% of all attributions, followed by powder (16.5%), pill (8.4%) and vinum (5.1%). Other preparations accounted for less than 4%. Wind was indicated in around a quarter of all preparations, followed by water (18.9%). Sleeping on heated herbs is attributed to wind element disorder diseases only. Of all vinum preparations, wind disorders account for almost half of the attributions.

**Table 2:** Attributions and preparations of the prescribed ingredients

**Attribution Preparation**	**All elements**	**Wind**	**Fire**	**Water**	**Earth**	**Antidote**	**Others**	**Total (%)**
Decoction (%)	3 (0.5)	117 (20.4)	128(22.3)	153 (26.7)	134 (23.3)	31 (5.4)	8 (1.4)	574 (62.8) (100)
Powder (%)	29 (19.2)	54 (35.8)	10 (6.6)	5 (3.3)	20 (13.2)	0 (0)	33 (21.9)	151 (16.5) (100)
Pill (%)	15 (19.5)	3 (3.9)	14 (18.2)	6 (7.8)	9 (11.7)	24 (31.2)	6 (7.8)	77 (8.4) (100)
Vinum (%)	4 (8.5)	21 (44.7)	7 (14.9)	0 (0)	0 (0)	0 (0)	15 (31.9)	47 (5.1) (100)
Sleeping on heated herbs (%)	0 (0)	32 (100)	0 (0)	0 (0)	0 (0)	0 (0)	0 (0)	32 (3.5) (100)
Taking juice (%)	0 (0)	0 (0)	2 (11.8)	6 (35.3)	2 (11.8)	2 (11.8)	5 (29.4)	17 (1.9) (100)
Boiling with meat (%)	0 (0)	2 (12.5)	5 (31.2)	3 (18.8)	6 (35.3)	0 (0)	0 (0)	16 (1.8) (100)
Total (%)	51 (5.6)	229 (25.1)	166 (18.2)	173 (18.9)	171 (18.7)	57 (6.2)	67 (7.3)	914 (100) (100)

### Clinical indications, diseases and symptoms

There were 277 clinical indications (111 diseases and 166 symptoms) mentioned in Book A. The most common indication (34.2%) was digestive system diseases. As mentioned, both disorders and prescriptions belong to wind problems. As illustrated in Table 3, among the eighteen most common symptoms, eight symptoms are related to gastrointestinal problems. The explanation of why TDM is more commonly used for treating digestive system problems was not mentioned in the book.

**Table 3:** Most common symptoms/indications of prescriptions

**Symptom**	**Frequency**
Abdominal pain^▴^	55
Fatigue/Lassitude	43
Soft or liquid stool^▴^	36
Weakness	33
Dark urine	29
Stomachache^▴^	29
Abdominal distension^▴^	29
Vomiting^▴^	29
Dry and hard stool^▴^	29
Dry mouth	28
No appetite^▴^	27
Yellow and greasy furred tongue*	25
White, greasy and thick furred tongue*	23
Pale face	23
Fever	23
Heart palpitation	20
Dizziness	20
Nausea^▴^	20

Note: * The symptom was probably influenced by the interpretation of TCM experts included in the textbook editing. ^▴^ The symptom related to gastro-intestinal disease.

Though we do not know how the editors of these textbooks bridge the gap between TDM and modern medicine, what is certain is that the philosophy of TDM is mainly symptomatic treatment. Some diseases, however, do not completely correspond with categorization of modern medicine. For example, in Book D, one disease called “Long Pi Le” by TDD includes all related woman’s postpartum symptoms and diseases. Another disease called “Long Niu” includes all related diseases of urinary infection and urolithiasis [7]. This kind of classification deserves further investigation.

### Herb species identification and plant parts

All ingredients mentioned in Book A have Dai names and/or Han names, and 70% of their botanical names could be identified in Book B which contain Han and Dai names as well as botanical names. Fourteen percent of the ingredients of prescriptions which could not be identified in Book B were available in a standard TCM book [8], while 12% could not be identified by any source. The remaining 4% were not herbs, and included honey, alcohol and chicken. TDD also adds various ingredients such as honey, rice wine, and rice-water as supplementary substances when prescribing treatment as well as fresh herbal juices. For example: the juice of "Huang Giu" (*Eclipta prostrata* L.), one commonly used herb, can be used as a supplementary substance as well as a principal ingredient in a prescription as well.

The prescriptions involved 388 herbs, 11 animals and 3 minerals. One hundred and forty six items were only mentioned once, and nearly 90% were used less than 6 times. The fifteen most commonly prescribed medicinal plants are listed in Table 4. The most commonly mentioned item was Biu Biu Liang (*Plumbago indica* Linn.) which appears in 24 (12%) of the 200 prescriptions.

**Table 4:** Most commonly used medicinal plants

**Local name**	**Botanical name**	**Attribution(s)**	**Application or indications**	**Preparation(s)**	**Frequency (%)**
Biu Biu Liang	*Plumbago indica* L.	Fire, wind and water	Whole herb used for arthritis/joint pain, abnormal menstruation, menstrual colic, cough, asthma, weakness, impotence.	Soup obtained from whole herb deposited in alcohol or after decoction to be taken orally.	24 (12.0)
Hao Ming	*Curcuma longa* L.	Water, earth and wind	Rhizome used for arthralgia, traumatic injury, distending pain of chest and abdomen, amenorrhea, irregular menstruation, swollen sores and furuncle, insect bites.	Soup or juice of Rhizome obtained after decoction or juicing to be taken orally.	20 (10.0)
Pi Nuai	*Piper nigrum* L.	Fire	Fruit used for abdominal pain, toothache, cough, asthma, diarrhea, nausea and vomiting.	Fruit made into powder or soup obtained from decoction to be taken orally.	19 (9.5)
Wen Shang Hai	*Arundina graminifolia* (D. Don) Hochr.	Water	Bulb and whole herb used for the deficiency of Qi and blood due to postpartum, poisoning of food, epilepsy, damp-heat jaundice.	Soup of bulb and whole herb obtained from decoction to be taken orally.	17 (8.5)
Guan Di	*Vitex trifolia* L.	Wind and water	Fruit and leaf used for dizziness, poor vision, hemiplegia, red swelling and pain of joints, spasm and pain of extremities, muscle and bone pain.	Soup of fruit or leaf after decoction to be taken orally.	17 (8.5)
Han Hao Nan	*Acorus calamus* L. var. verus L.	Wind, water and earth	Rhizome used for abdominal pain and diarrhea, abdominal distension, nausea and vomiting, dizziness and headache, insomnia and dreamful sleep, asthma.	Soup of rhizome obtained from decoction to be taken orally.	16 (8.0)
Jing Lang	*Nigella glandulifera* Freyn et Sint.	Wind and water	Seed used for dizziness, headache, insomnia and dreamful sleep, postpartum bleeding, anemia, hypogalactia, menoxenia, menstrual colic, amenorrhea, epilepsia, muscle and joint pain, stomachache.	Chewed seed to be taken orally or smashed seeds to be sniffed.	15 (7.5)
Mi Huo Wa	*Tacca chantrieri* Andre’	Fire, water and wind	Rhizome and leaf used for mumps, submaxillary lymph nodes soreness, galactophore soreness, sore throat, cough, phlegm much, abdominal pain and unknown toxin.	Soup of rhizome or leaf obtained from decoction to be taken orally.	15 (7.5)
Guo Long Liang	*Cassia fistula* L.	Wind and water	Fruit, root, stem, bark and leaf used for oppilation, dysuresia, urinary stones, frequent and scanty urination with continuous dripping, sore throat due to heat-wind evils, orolingual furuncles, tumor, headache, dizziness.	Fruit to be chewed and taken orally. Soup of root, stem, bark and leaf obtained from decoction to be taken orally.	14 (7.0)
Huang Giu	*Eclipta prostrata* (L.)L.	Water	Whole herb used for dysentery, abdominal pain, febrile convulsion, skin and external diseases.	Juice extracted from whole herb to be taken internally or applied externally.	13 (6.5)
Zhu Zha Ling	*Tinospora sinensis* (Lour.) Merr.	Water	Vine used for insufficiency of Qi and blood, general debility and lassitude, palpitation, pain of limbs and joints due to wind-dampness evils, blood stasis and swelling due to traumatic injury.	Vine to be deposited in alcohol or made into powder and the alcohol or powder to be taken orally.	13 (6.5)
He Tao Leng	*Spatholobus suberectus* Dunn.	Water, earth and wind	Vine used for dysmenorrhea, amenorrhea, postpartum abdominal pain, and anemia and to reduce rheumatic pain and joint aches.	Soup of vine obtained from decoction to be taken orally.	12 (6.0)
Bu Luei	*Zingiber purpureum* Rosc. or *Zingiber corallinum* Hance.	Earth	Rhizome used for abdominal pain, abdominal distension due to food retention, nausea and vomiting, hepatosplenomegaly, arthralgia due to wind-heat.	Soup of rhizome obtained from decoction or powder of rhizome to be taken orally.	12 (6.0)
Ya La Meng	*Cassia obtusifolia* L.	Water	Whole herb used for insomnia and dreamful sleep, cramps, jaundice, malaria, hepatitis.	Soup of whole herb obtained from decoction to be taken orally.	12 (6.0)
Hiam Leng	*Mahonia bealei* (Fort.) Carr.	Earth and water	Stem used for jaundice, abdominal distension, heat and pain, dry mouth and throat, diarrhea, orolingual sore, irritability, menorrhagia.	Soup of stem obtained from decoction to be taken orally or applied externally.	11 (5.5)

It was noted that underground plant parts, such as root, rhizome, tuber, groundnut and bulb, were all designated by a single term: “Ha” meaning root. The most commonly used plant part was the root accounting for 38.2%, followed by whole herb (19.6%) and stem (12.1%). Other medicinal parts were used in less than 6% of plant parts. (Table 5)

**Table 5:** Medicinal parts of plants (n = 402)

**Medicinal part**	**Frequency (%)**
Root	349 (38.2)
Whole herb	179 (19.6)
Stem	111 (12.2)
Seed	55 (6.0)
Fruit	47 (5.1)
Leaf	43 (4.7)
Bark	40 (4.4)
Pith	14 (1.5)
Flower	20 (2.2)
Latex	12 (1.3)
Bud	11 (1.2)
Other	33 (3.6)
Total	914 (100)

Note: the total is 914 because some herbs can be used in more than one medicinal part for medical purposes.

### Dosage

The same herb used in different prescriptions had different dosages. For example, the dosage of Biu Biu Liang varied from 5 g to 500 g. The different dosages may indicate different roles of the same plant in different prescriptions.

## Conclusion

Our analysis has summarized the nature of TDM theory which was expressed in prescriptions, attributions and indications. While commonly used herbs and their indications have been ascertained, the theories behind TDM need further studies.

### Limitations

Han (Chinese) words used in the textbooks often have different pronunciation from Dai. Terms and concepts translated might be distorted by the influence of TCM prevailing in China.

## Competing interests

The authors declare that they have no competing interests.

## Authors’ contributions

ZC collected and analyzed the data and wrote the manuscript. VC and ZC designed the study. VC, NK and YL supervised and provided comments on the draft of manuscript. All authors read and approved the final manuscript.
